# Extended genomes: symbiosis and evolution

**DOI:** 10.1098/rsfs.2017.0001

**Published:** 2017-08-18

**Authors:** Gregory D. D. Hurst

**Affiliations:** Institute of Integrative Biology, University of Liverpool, Liverpool L69 7ZB, UK

**Keywords:** symbiosis, heredity, extended evolutionary synthesis

## Abstract

Many aspects of an individual's biology derive from its interaction with symbiotic microbes, which further define many aspects of the ecology and evolution of the host species. The centrality of microbes in the function of individual organisms has given rise to the concept of the holobiont—that an individual's biology is best understood as a composite of the ‘host organism’ and symbionts within. This concept has been further elaborated to posit the holobiont as a unit of selection. In this review, I critically examine whether it is useful to consider holobionts as a unit of selection. I argue that microbial heredity—the direct passage of microbes from parent to offspring—is a key factor determining the degree to which the holobiont can usefully be considered a level of selection. Where direct vertical transmission (VT) is common, microbes form part of extended genomes whose dynamics can be modelled with simple population genetics, but that nevertheless have subtle quantitative distinctions from the classic mutation/selection model for nuclear genes. Without direct VT, the correlation between microbial fitness and host individual fitness erodes, and microbe fitness becomes associated with host survival only (rather than reproduction). Furthermore, turnover of microbes within a host may lessen associations between microbial fitness with host survival, and in polymicrobial communities, microbial fitness may derive largely from the ability to outcompete other microbes, to avoid host immune clearance and to minimize mortality through phage infection. These competing selection pressures make holobiont fitness a very minor consideration in determining symbiont evolution. Nevertheless, the importance of non-heritable microbes in organismal function is undoubted—and as such the evolutionary and ecological processes giving rise to variation and evolution of the microbes within and between host individuals represent a key research area in biology.

## 1. Introduction

It has long been understood that many aspects of a multicellular organism's biology are underpinned by the symbiotic microbes with which it interacts [1,2]. In plants, mycorhizzal fungi determine competence to uptake micronutrients from soil, *Rhizobia* bacteria in nodules make atmospheric nitrogen available to the host in the form of ammonium, and resistance to herbivory may be determined by the fungal endophytes that reside on leaves [3]. In humans, approximately 50% of the cells in the body are microbial [4], and microbes within the gut directly digest otherwise inaccessible plant-derived complex polysaccharides, making this nutrition source available to the animal in the form of short-chain fatty acids. Gut microbes may also enable mammals to live on otherwise toxic diets: some plant material may become accessible to animals by virtue of microbial degradation of otherwise toxic plant secondary compounds [5]. In insects, the majority of species carry heritable bacteria that live inside cells of the host and pass to progeny through eggs. Many aspects of individual biology—from amino acid and vitamin anabolism through to protection against natural enemies—derive from these symbionts [6–8]. These examples (and others) lead to the realization that plants and animals live in a microbial world [1,2].

The individual-level impacts of microbes described above are reflected in symbiotic microbes' impact upon the ecology of many species [1]. Indeed, in many cases the niche of an organism is partly derived from the capacities given to the individual by virtue of the microbes it is associated with. Where a plant can grow is dependent on its ability to take micronutrients from the soil via fungal associates (e.g. mycorrhizal associates of ericaceous plants). That many insects live by sucking phloem (a diet depauperate in amino acids) and blood (a diet lacking B-vitamins) commonly requires the biosynthetic capacity of symbiotic microbes [9–11]. Insect use of cellulose or lignin-based diets is heavily dependent on microbial degradation of these polysaccharides [12]. Some of these niche usage properties occur at a clade level: aphids as a group can live on phloem by virtue of symbiosis with *Buchnera* microbes that synthesize tryptophan [13]. Thus, associations with microbes define the niche of many animal and plant species.

The importance of microbial associates in determining individual phenotype led Lynn Margulis to develop the concept of the holobiont—the individual as including microbial symbiotic associates [14]. After a period where the term was rarely used outside of symbiosis research, the concept has been revitalized alongside the in-depth study of microbiomes afforded by high-throughput sequencing, and the realization of the profound impact microbial associates have on organismal function outside of ‘classical’ symbiotic models. The holobiont concept has recently been more fully developed [15–18] and subsequently clarified [19]. For instance, the term hologenome is used to reflect the sum of all genomes comprising the holobiont, creating equivalence between ‘host’ genes and symbionts within the individual. The concept has not proved uncontroversial [20,21]. For instance, Queller & Strassmann [22] examined features creating ‘organismality’—that is to say, when to consider entities with previously independent evolutionary histories as one unit. They conclude that organismality requires domination of cooperation of the entities with respect to individual fitness—that the ‘organism is not much disrupted by adaptations at lower levels'. This creates a much more stringent view of individuality than the holobiont concept.

In this review, I first investigate one particular question—the degree to which the holobiont represents a level of selection. The idea of the holobiont as a unit of selection is captured by the opening sentence of Rosenberg & Zilber-Rosenberg [17, p. 1]:

The hologenome concept of evolution postulates that the holobiont (host plus symbionts) with its hologenome (host genome plus microbiome) is a level of selection in evolution.

Following discussion of this question, I outline ways in which the evolutionary ecology of traits encoded by heritable microbes differs from a ‘standard’ model of nuclear encoded traits, and then examine how we may understand the evolution of polymicrobial communities where community composition has a heritability, but direct inheritance of the microbes is lacking.

## 2. The holobiont as a unit of selection

To dissect the idea that the holobiont is a unit of selection, we can start by examining a simplified verbal model of natural selection and adaptation. For a standard trait, this can be stated as three ‘ifs’ and a ‘then’:

— if (a) there is phenotypic variation in a character,
— and (b) this variation is associated with variation in the probability of survival/ fertility between individuals (fitness variation),
— and (c) there is heritability to the phenotype (genetic variation)

Then, the composition of the next generation will be biased with respect to heritable factors that increase the probability of survival/fertility.

Applying this ‘evolution 101’ approach to microbial symbiont associates, we can posit that if (i) there is between-individual variation in microbial associates that produces phenotypic variation and (ii) some of this variation is associated with variation in the chance of survival/reproductive capacity of the host and (iii) there is a heritable element to microbiomes, then selection will occur on microbiome constitution.

### 2.1. Phenotype and fitness variation associated with variation in microbial symbionts

We can assess the first two aspects required for natural selection to act together. There is abundant evidence that between-individual variation in microbial associates exists, and that this variation is associated with biological differences that translate into holobiont fitness variation.

Between-individual variation in microbial associates/fitness can occur at multiple levels. At the most basic level, there may be a symbiont ‘species’ that is present in all hosts within a species, but the symbionts that are present are genetically variable between-individual hosts, and this variation produces differences in host fitness and phenotype. There are a variety of examples described in insects. Pea aphids vary in the *Buchnera* strains they possess, with genetic variation at the *Buchnera* ibpA gene associated with variation in tolerance of the aphid to heat shock [23]. Similarly, aphids carrying different strains of the symbiont *Hamiltonella* vary in susceptibility to parasitic wasp attack [24]. Here, there is the added complexity that aphid genotype and parasitic wasp strain also combine to determine this phenotype (see [25] for review). Both of these microbe-variant associated traits are clearly also associated with fitness variation, in the case of *Buchnera* during hot summer days, and in the case of *Hamiltonella*, in the presence of the natural enemy.

The second level of variation is in the presence of a particular symbiotic microbe. In arthropods, it is common for particular endosymbionts (symbionts existing in the body of the insect, rather than the gut or external epithelia) to be present in some individuals and absent in others (e.g. [26]). For instance, around 40% of *Drosophila hydei* individuals in natural populations carry a *Spiroplasma* symbiont in their haemolymph [27]. Individuals with the *Spiroplasma* are resistant to attack by some parasitic wasps [28,29], and thus presence/absence variation of the *Spiroplasma* affects fly fitness.

Finally, in polymicrobial communities, there may be between-individual variation in microbial community composition. Here, when microbial communities are sampled, they are found to vary either in the taxa present, or (more commonly) in the relative frequency of members of different taxa. In humans, initial analysis suggested the existence of ‘enterotypes’—the microbial communities found within guts of different individuals cluster into three main community types [30]. While the presence of discrete clusters is under debate, the existence of inter-individual variation is not. It is also likely that current methods underestimate total inter-individual variation. Traditionally, 16S rRNA sequencing is used to assign sequences into operational taxonomic units. A single operational taxonomic unit determined by the sequence of this slowly evolving gene may within itself capture (or obscure) a large amount of microbial diversity [20]. Our knowledge of variation in these communities is thus much less sophisticated than our understanding of equivalent variation between-individual nuclear genomes, and will underestimate the variation that exists. The phenotypic impacts of variation in polymicrobial communities are harder to gauge than for binary symbioses, because there is substantial developmental and other temporal variation in microbiome constitution within an individual (e.g. [31]). Nevertheless, transplantation experiments in *Peromyscus* deer mice indicate that the native gut microbiota, or that from closely related species, is more efficient in terms of digestion than microbiota from more distantly related species [32]. Thus, while associations of fitness with microbiome constitution are complex, it is clear that there are important phenotypic consequences of microbiome variation.

In summary, it is undoubtedly true that inter-individual variation in the microbiome exists. It takes the form of variation in symbiont genotype in binary associations, variation in the presence/absence of symbionts in binary associations, and variation in the representation of symbionts in polymicrobial microbiome communities. It is also clear that inter-individual variation in symbiont partners is associated with variation in fitness.

### 2.2. Heritability of symbiont presence

The last criterion for selection to act is that parents with particular microbiome variants are more likely than average to produce progeny with similar microbiome variants, such that fitness variation leads to biased transmission (i.e. selection occurs). Here, it is important delineate two means by which parent–offspring resemblance in microbiomes may occur. The first is through *direct* heredity of the microbe—that is to say, the microbe individual/lineage in the progeny is a direct descendent of that in the parent. The second I will call *indirect* heredity. This is where the progeny of a particular individual carry similar microbial isolates/consortia to their parent by virtue of genetic variation in the host nuclear genome that impacts on the identity of the microbes that then colonize the progeny [33]. Under indirect heredity, the microbes in progeny are not transmitted directly from the parent, but are environmentally acquired. Variation in microbiota here is associated with host nuclear genetic variation, which may be a result of selection on the host with respect to the establishment of the microbial community, or microbes with respect to establishment on different host genotypes (or both).

### 2.3. Direct inheritance of symbionts

Direct inheritance of symbionts is very common. Vertical transmission (VT, transmission from parent to offspring) of symbionts occurs ‘de facto’ for intracellular symbionts within single-celled organisms, as cell division partitions the symbiont population (generally a clone) into each fission product. VT of symbiotic microbes is also very common in fungi and plants, where a variety of viruses, bacteria and (in plants) fungi show direct parent–offspring transmission (table 1). Animals commonly show VT of symbionts—over a half of insect species carry heritable bacteria [26], and members of this group also carry an array of heritable viruses, single-celled eukaryotes and fungi [41,46] (table 1). VT of symbionts is also seen in vertebrates (e.g. [47]).

**Table 1.** Heritable microbe diversity in plant, invertebrate animal and fungal hosts.

host	symbiont	example (symbiont, host)	symbiont transmission	phenotype	reference
plant	virus	pepper cryptic virus-1 in *Capsicum annuum*	biparental, through pollen + ovules	not known	Arancibia *et al*. [34]
	bacteria	*Cand. Burkholderia kirkii* on *Psychotria kirkii*	maternal, on seed	probably defensive, also required for plant development	Carlier & Eberl [35] and Miller [36]
	fungi	*Epichloe festucae* on cool season grasses	maternal on seed	defensive against herbivores	Schardl [37]
fungus	virus	CThTV in *Curvularia protuberata*	in conidiospores	thermotolerance	Márquez *et al*. [38]
	bacteria	*Burkholderia* in *Rhizopus microsporus*	in spores	offensive toxin production; required for sporulation	Partida-Martinez & Hertweck [39] and Partida-Martinez *et al*. [40]
invertebrate animals	virus	Sigma virus in *Drosophila melanogaster*	biparental in eggs	deleterious; CO_2_ sensitivity	Longdon & Jiggins [41]
	bacteria	*Wolbachia* wRi in *Drosophila simulans*	maternal in eggs	anti-viral tolerance; cytoplasmic incompatibility	Hoffmann *et al*. [42] and Osborne *et al*. [43]
	fungi	*Leucoagaricus* in *Acromyrmex* ants	maternal, carried by foundress queen ant	nutrient provisioning; external gut	Chapela *et al*. [44]
	unicellular eukaryotes (e.g. microsporidia)	*Ambylospora* sp. in *Culex salinarius*	maternal, within eggs	parasitic, kills male hosts for infectious transmission	Andreadis & Hall [45]

VT in species with separate sexes is commonly maternal, with transmission exclusively from mother to progeny. Endophytic fungi in cool season grasses, for instance, pass from maternal plant soma onto seed, but do not transmit via pollen [3]. The majority of heritable bacteria in insects pass through eggs only, associated either with anisogamy restricting transmission through sperm (but see [48]), or through maternal–egg contact during oviposition. By contrast, biparental transmission does occur commonly for viruses (e.g. sigma virus in flies, many viruses in plants) [41,49]. Here, maternal transmission is commonly more efficient than paternal, but both exist at significant levels. There are also a variety of cases where viral, microsporidial and bacterial symbionts combine vertical and infectious transmission (e.g. [50–52]).

While more sporadically observed, there may also be direct heredity of polymicrobial consortia. Marine sponges, for instance, host a diverse range of microbial symbionts. Commonly, each species has a microbiota distinct from others, and the microbes while present in the environment at low levels are found predominantly in symbiosis with sponges. Many sponge species are viviparous—that is to say embryos develop within the body of the mother. The microbial consortia are then directly transmitted from parent to offspring [53–56]), although it is currently unclear the extent to which it persists to adulthood [57]. Similar consortium transmission may occur during birth in humans [58]. Again, the degree to which these microbes persist through host development to establish multigenerational transmission is uncertain.

The presence of VT for symbionts varies between host taxa, symbiont taxa and environment. For plants, VT is rare for root-associated microbes, but more common for above-ground symbionts, presumably associated with access to forming seeds [59]. Parent–offspring transmission is apparently more common in terrestrial host taxa than marine (although marine examples do exist, e.g. sponge examples above, also clams: [60]); this distinction may be associated with reduced ability of symbiotic microbes to survive and transmit in the harsh non-host terrestrial environment. VT of symbionts is more common in invertebrates than vertebrate animals, potentially associated with differences in immune system, in particular, the increased capacity for detection of intracellular microbes by vertebrates. Within insects, VT is more common for members of Gram-negative bacterial clades than Gram-positive [61]. The dearth of heritable Gram-positive microbes may be associated with the capacity of Gram-positives to survive in the environment through spores, or to properties of a cell wall that make intracellular life less likely. It is notable that the one major lineage of Gram-positives to be vertically transmitted commonly are the Mollicutes, which have secondarily lost their cell wall and produce no measureable immune reaction in insect hosts even when extracellular [62].

Direct heredity of symbionts may be driven in three ways. First, it may be the microbe that encodes the capacity to enter the germ line of the host and thus transmit to the next generation. In insects, heritable microbes have multiple means of entering ovaries (e.g. [63,64]), and it is clear from transinfection experiments that these mechanisms work in different host species (although host background clearly does affect the ability of the microbe to enter the germ line). Second, the host may physically transfer the microbe to progeny. In aphids, for instance, *Buchnera* symbionts move to the next-generation within-host bacteriocyte cells that fuse with embryonic material [65]. In other insects such as stinkbugs, microbial capsules are deposited on the egg, and are then consumed by the hatching larva, which thus obtains the direct descendents of their mother's symbionts [66]. Transfer may also be direct through carriage (for fungal gardens of leaf cutter ants) or through parent–offspring feeding (proctodeal trophylaxis for termite hind gut symbionts) [67]. Finally, VT can occur without ‘action’ on the part of either symbiont or host through simple proximity of parent and offspring. Where parental care exists, for instance, the contacts that occur between parent and offspring, and the shared immediate environment, are likely to produce direct transmission of symbionts (see [47] for review).

### 2.4. Indirect heredity of symbionts

As described above, indirect heredity of symbionts is the tendency of parent and offspring to share symbiont variants/symbiont species/microbiome assemblages *without* direct transmission of the symbionts from parent to offspring. In this process, hosts assort with different symbiont by virtue of variation in their nuclear genome, and microbes may assort with hosts likewise. The inheritance of variants in the nuclear genome then drives the association between parent and offspring in the symbiotic microbes present.

There is abundant evidence for indirect heredity of this kind (see [33] for review). In humans, twin studies provide evidence in the form of greater resemblance of gut microbiome constitution of identical twins compared to fraternal [68]. In *Drosophila melanogaster*, different isogenic lines predictably establish different microbiomes from a common exposure pool [69], again establishing intra-species genetic variation for microbiome assembly. More widely, there is abundant evidence that there is selectivity in host–symbiont interactions, for instance, mediated through the innate immune system and pattern recognition receptors [70]. Evolution in PRR systems may be driven by the particular symbiosis, or through interaction with other pathogens/commensals.

This evolution may ultimately lead to the interspecific differences observed in microbiome assembly. For instance, the different microbiomes established in different *Hydra* species are associated with differences in the antimicrobial peptide complement [71]. Evolutionary divergence of these systems between species can create the pattern of phylosymbiosis [72,73], where microbial community composition depends on host species, with higher concordance between related species [32,74]. Indeed, hybrid inviability can be microbe-dependent, indicating integration of microbiome assembly and host factors [73].

### 2.5. Direct heredity and selection at the level of the holobiont

With direct heredity, the fitness of the symbiont is strongly correlated to that of the ‘host’ individual. Where the individual carrying the symbiont dies more often, or fails to reproduce more commonly, or reproduces more poorly than individuals either not carrying the symbiont, or carrying a different variant, then the symbiont/symbiont genotype declines in frequency in the population. Thus, the fate of the symbiont rests strongly on the fate of the individual that can carry and transmit it.

This logic has historically been implicit in models of heritable symbiont dynamics. The ‘basic formulation’ treats symbiont variation as genetic variation, and models changes in frequency over time in relation to the impact of the symbiont on host fitness (e.g. [75,76]). These models have transmission parameters that differ from Mendelian genetics (most commonly maternal transmission with segregational loss), but are in other regards conceptually equivalent to population genetic formulations. The logic has also been explicit in many verbal treatments—maternally inherited symbionts are selected to maximize the fitness of their female host, measured in terms of the number of infected daughters produced.

The statement that heritable symbionts are selected at the level of host individual is not equivalent to stating this is the only level of selection. It is well recognized that maternally inherited microbes have no evolutionary interest in male hosts, as these hosts cannot transmit them [77]. Thus, selection can act in two non-mutually exclusive directions—to increase female host survival and reproduction through contributing to individual function, and to increase either the production or survival of daughters through manipulation of sex allocation or male/female survival. These latter traits, collectively known as ‘reproductive parasitic phenotypes', are in opposition to fitness of the host individual. Indeed, some symbionts combine sex ratio distortion and host beneficial traits (e.g. [78]).

Further to this conflict, heritable microbes do not exist under the strictures of ploidy of nuclear genes. There may be many individual symbionts within both a cell and a host, and a fraction of these are transmitted to the next generation. Importantly, there is commonly soma to germline movement (which contrasts with the more segregated germ line of nuclear encoded elements in animals). This large population size of symbionts shrinking to fewer transmitted symbiont individuals leads to the possibility of within-host selection, favouring types that are over-represented in the transmission gene pool. This type of selection is expected to be most common where there is biparental inheritance or additional infectious transmission that creates mixing of symbiont lineages. However, it has also been hypothesized to occur for maternally inherited elements [79].

Pragmatically, noting that the holobiont is a unit of selection in the case of heritable microbes produces no conceptual change to our understanding of multilevel selection; we have always understood (in past treatments) that heritable symbionts are selected in part to maximize the survival and reproduction of their host, and this occurs notwithstanding intragenomic conflict with respect to the production and survival of male hosts, and potential selection with respect to competition within hosts. It is arguable that the individual host is the predominant level of selection for heritable microbes.

### 2.6. Indirect heredity and selection at the level of the holobiont

Under *indirect heredity*, the importance of the holobiont as a level of selection is less clear. Indirect heredity by definition removes host reproduction as a fitness-related character for the symbiont, as the symbiont is not transmitted through host reproduction. Symbiont transmission is through infectious transmission, and the primary fitness-related trait for the symbiont thus lies in this transmission pathway.

Where a symbiont lineage associated with a host is clonal and persistent (commonly referred to as symbioses with *partner fidelity*: [80]), symbiont fitness does remain strongly associated with host survival, so long as the symbiont gains transmission from the host (rather than being exploited/captured, e.g. [81]). For instance, bobtail squid are colonized by *Vibrio fischeri* symbionts which are housed in the light organ. Squid and *Vibrio* represent a highly integrated symbiosis, with codevelopment and selectivity (by both microbe and host; see [82] for review). Within this symbiosis, the symbiont population undergoes diurnal patterns of growth within the light organ followed by expulsion of 90% of the symbiont population into the water (for the *Vibrio*, this is transmission) [83]. The symbiont has an evolutionary interest in the survival of the bobtail squid, as the longer the host lives, the more daily expulsion events occur and the greater the transmission. Indeed, *Vibrio fischeri* aids this survival by providing its host with bioluminescent countershading.

In many cases, however, the host-associated microbiome is diverse—both different strains of particular bacteria and different bacteria within a host individual. For instance, while human guts commonly carry a dominant *E. coli* strain, this is always in the context of other co-occurring strains of *E. coli* (and of course, many other bacteria). The strains present may be subject to turnover, such that a particular *E. coli* individual in the gut in 1 year is not necessarily a direct descendent of those found previously (see [84] for review). More widely, Faith *et al*. [85] examined the temporal stability of the human gut microbiome. Analysis using the 16S rRNA gene sequence indicated a high level of stability of taxa within the community over 5 year sampling, with more than half being retained. More refined analysis using whole-genome sequencing of cultured isolated indicated less stability at the individual strain level. Nevertheless, 36% of strains were isolated from multiple time points, indicating that the interactions can persist over years. Outside of humans, studies of sponges have shown resilience of microbiota composition to short-term environmental changes [86].

In summary, partner fidelity for microbes in polymicrobial communities is less profound than in binary interactions (at least from the symbiont standpoint). Under these circumstances, the evolutionary interest of the microbe in host survival may be lessened. Where there is little fidelity, the symbiont has little ‘interest’ in the long-term survival of the host. Furthermore, the turnover of polymicrobial communities strengthens the relative importance of other determinants of microbial fitness—the ability to outcompete other strains and species in the community; the ability to resist virus attack, the presence of other strains which benefit the microbe through by-product use, the ability to resist any host immune activity; the ability to stay inside the host while maintaining dispersal of progeny through faeces. Consistent with this, experimental studies indicate microbiota composition is a major modulator of the intensity of selection in gut *E. coli* [87].

At this point of complexity, it appears that the holobiont as a level of selection is, pragmatically, not central for understanding the diversity or evolution of holobionts. To understand the individual and its microbial community, we need to understand community assembly and the ecological dynamics of microbial communities, the role of evolution within the host in driving changes in the microbiome, the variation in diet/other conditions that may favour different strains. We also need to understand the nature of selection on the host to control overall community assembly and dynamics, as this assembly is an important fitness determinant for the host and likely drives the observations of phylosymbiosis that allows coadaptation and coevolution. The interplay between these forces then determines the diversity and evolution of holobionts.

Nevertheless, it has been argued that VT is not necessary for symbionts to be equivalent of genes. Fitzpatrick [88] notes that covariance between parties is the core requirement for the parties to be considered in a single conceptual framework, and while this covariance is generated through VT, it may also occur with horizontal transmission. Covariance may be generated by spatial correlations, or if there is significant epistasis between microbe and host that generates covariance between the two parties. Fitzpatrick concludes:

It does not necessarily follow that host-symbiote systems should be conceptualized as ‘meta-organisms,’ but the theoretical continuity between coevolution of genes within genomes and between genomes is encouraging for further synthesis between evolutionary genetics and evolutionary ecology. [88, p. 11]

Within this conceptual framework, partner fidelity and microbe–microbe interactions nevertheless remain central to the equivalence of symbiont and genes. Reductions in fidelity, and increases in the importance of microbe–microbe interactions in polymicrobial communities, are expected to reduce associations and disrupt the holobiont as a ‘unit of selection’.

A further examination of this issue derives from Doolittle & Booth [89], who envisage ‘metabolic and developmental interaction patterns’ as units of selection. This treatment provides a major contrast, in that it ignores the fitness interests/transmission of the parties, with a focus instead on the host. Within this, the identity of the identities of actual symbiont parties is less important than their functional competencies. The holobiont is defined by the interactions the host has been selected to create in terms of the functional competencies of microbes (an overall biochemical/physiological phenotype), rather than the particular parties involved and their fitness interests. This is an interesting conceptual extension to our views on host evolution and its contribution to variation in, and evolution of, host-associated microbiota. However, the framework uses the ‘levels of selection’ concept rather differently to common usage in evolutionary biology.

## 3. Evolutionary patterns for traits encoded by heritable microbes

Above it was argued that heritable symbionts can in many senses be treated ‘as genes’ in terms of spread through populations. However, this equivalence does not make symbiont-encoded traits direct evolutionary equivalents of traits encoded in the nuclear genome [90]. Three distinctions in the evolutionary ecology of heritable symbiont-encoded traits have been made.

### 3.1. Symbiont-encoded variation is very dynamic

The magnitude of the selective coefficient associated with presence/absence symbiont-encoded variation is commonly greater than that observed for genetic variation at nuclear loci. First, because there is an energetic cost to carrying a large number of symbiont cells within the host, it is likely to be rare that symbiont present and symbiont uninfected individuals are ever equivalent in fitness terms. For instance, *Pseudonocardia* heritable symbionts increase the basal metabolic rate of its ant host by 10% [91]. Second, the magnitude of benefit associated with symbiont presence (their drive) may be large. While subtle effects are possible, many of the traits encoded by microbial partners have strong fitness impacts; defence against natural enemies, for instance, provides very strong selective benefits when the natural enemy is common.

In addition, many heritable symbionts show imperfect maternal inheritance, wherein only a fraction (typically 95–99%) of progeny inherit the infection [92,93]. This segregational loss also leads to declines in the frequency of the symbiont in the absence of a ‘drive’ benefit that increases female host fitness. Furthermore, where the rate of segregational loss is environmentally contingent, this may be reflected in seasonal dynamics [94]. Together, these observations lead one to conclude that symbiont present/absent variation is unlikely to be subject to drift processes. A symbiont will either exist at a balanced polymorphism against uninfected cytotypes, or decline through selection, or increase through selection.

Laboratory experiment and observations of natural environment dynamics confirm this logic. In laboratory culture, heritable symbionts commonly increase rapidly in frequency in the presence of their ecological driver, and decline in the absence of the driver (e.g. [95]). In the field, many examples of rapid adaptation are associated with symbiont spread/loss. For instance, heritable *Rickettsia* spread through *Bemisia* white fly populations in the USA occurred in a 10 year period [78]; heritable *Spiroplasma* infections that confer tolerance of nematode infection occurred in a 20 year period [96]. Rapid losses are also seen in natural populations, especially in relation to seasonal dynamics [97]. These observations fit a model where the magnitude of the selective coefficient—either benefit or cost—is high.

Heritable symbiont dynamics may also commonly require an eco-evolutionary framework. Where heritable symbiont traits alter the outcome of biotic interactions, they then alter the dynamics of the interacting parties. The presence of a defensive symbiont, for instance, increases mortality of the natural enemy against which it protects. Thus, the dynamics of these systems can be predicted only from modelling the tritrophic interaction, include enemy dynamics [98].

### 3.2. Symbiont-encoded traits have different ‘mutational’ origins

Within the evolutionary process, mutation is the ultimate source of novelty. Mutation here is defined in the broadest sense of any heritable changes. For classical nuclear encoded traits, mutation may be in gene sequence or gene regulation; it may also be the creation of novel genes. For heritable symbiont traits, mutation events may be similar—a DNA-based change in an existing symbiont (that alters the property of that symbiont), creating variation between symbiont strains circulating in the population. Alternately, the mutation may be the presence of a novel heritable symbiont, creating presence/absence variation.

In one model, new heritable symbiont–host interactions may evolve through the evolution of inheritance of a symbiont from an existing non-heritable symbiosis. This may occur if the symbiont evolves to enter and transmit through the germ line, or if the host evolves to transmit the symbiont to progeny. However, analysis of the relatedness of symbiont strains across host species indicates that the majority of novel heritable symbiont–host combinations arise following host shift events, where a heritable microbe in one host species moves into another host species. In this regard, heritable microbes are biologically similar to plasmids in bacteria. For instance, an ectoparasitic mite can feed off a *D. nebulosa* carrying *Spiroplasma*; if it then moves to feed upon a *D. melanogaster* individual, the *Spiroplasm*a may transfer to that new host species and be vertically transmitted in it, using its established genetic machinery [99].

Host shift events represent a major mutational event from the viewpoint of the host—the introduction of a new compartment of heritable variation [100]. They are also, in a sense, a major event for the symbiont—a new (and likely rather different) host environment. The introduction of a new heritable symbiont will commonly represent a mutation of large effect—commonly deleterious and unable to propagate (e.g. [101]), but sometimes introducing well-formed adaptations (such as natural enemy protection) to the host (e.g. [102]).

Importantly, host species may vary in their likelihood of a novel heritable symbiont being introduced. This variation may derive from biological factors such as immune system function. For instance, it is thought spiders may commonly have heritable microbes because they lack one of the two major innate immunity pathways, that centred on the imd (immunodeficient) locus. The likelihood of acquisition may additionally depend on the community context—host shifts occur most commonly between species that are ecologically proximate (e.g. [103]), and may be more likely to create compatible combinations where the recipient species phylogenetically related to the ancestral host (e.g. [104,105]).

### 3.3. Symbionts represent non-recombining elements subject to mutational decay through Muller's ratchet

Within their host context, heritable microbes become isolated from genetic exchange, may evolve to lose the genetic machinery for recombination, and also be subject to small population size during VT [106]. These events can lead to the fixation of deleterious mutations through drift without the capacity for recombination to reconstitute the ‘fit’ form. This process of Muller's ratchet accumulation may degrade heritable symbionts over time, such that they become obligately dependent on their host species (i.e. lose capacity to host switch), require host factors for their maintenance, and may ultimately become inviable. While this process may take several million years, it suggests heritable symbionts may present an ‘evolutionary rabbit hole’ for a host—a path more easily entered than extricated from [79]. For instance, aphids may be limited in their geographical range by the thermal tolerance of their required *Buchnera* partners, because mutational decay has rendered *Buchnera* proteins highly heat-sensitive.

Further to these distinctions from evolutionary ecology of ‘genes’, it is notable that the heritable symbiont phenotype may have a transgenerational ‘epigenetic’ component associated with symbiont titre. The number of symbiont cells within a host individual (titre) can affect both the strength of phenotypic change experienced by the host, and the fidelity of symbiont transmission. Importantly, low titre infections in a female tend to produce low titre infection in the progeny, a transgenerational influence [107]. In parallel to many epigenetic impacts on DNA sequence in animals, this historical influence will be unlikely to persist across multiple generations, but may nevertheless influence the dynamics of the symbiont.

## 4. Conclusion

Heritable microbes represent important elements of individual biology in many taxa. The direct inheritance of the agent means their evolutionary biology can be approximated in a population genetic framework. However, as argued above, there are interesting and important distinctions in terms of the evolutionary ecology of symbiont-encoded traits.

For cases where the microbiome has indirect heredity, the evolutionary and ecological processes defining the microbiome are complex. Indeed, the importance of microbial associates in defining individual phenotype makes understanding the assemblage, dynamics and evolution of complex microbiomes one of the most pressing challenges in understanding between-individual variation. The observations of phylosymbiosis also imply evolutionary diversification of assemblage processes at some level, either under direct selection on the host to assemble ‘host-friendly communities', or through indirect selection on the host to avoid pathogens that then impact on microbiome assembly, or through selection on microbes to colonize particular types of host.

The microbiome is well recognized as ecologically dynamic. Changes in diet shift the community composition towards those microbes most able to use the diet [31]. What is less well recognized is that it is likely also very dynamic in evolutionary terms. Experimental evolution studies have repeatedly demonstrated that the large population size and short generation time of microbes makes them evolutionarily labile. For vertebrate gut communities of large size, it is highly likely that evolution occurs commonly within the individual, altering the phenotype of the host individual. Barroso-Batista *et al*. [108] demonstrated evolution of *E. coli* colonizing the mouse gut through recurrent soft sweeps. Remarkably, the pattern of evolution was highly repeatable, with adaptation of the microbe to the gut taking the same pathways.

One area of recent interest is in the role of evolution of microbes within guts in protecting the host against pathogen attack. King *et al*. [109] have recently demonstrated how probiotic behaviour—a microbe defending its host against invading pathogens—can evolve in the context of the gut through selection on one microbe to defend itself against a competitor. The trait of host defence is not selected for at the holobiont level (microbes were derived from dead hosts during the experiment)—but nevertheless is adaptive at this level. Thus, an individual's ability to clear or tolerate infection is likely a product both of its own immune system and the ecological and evolutionary dynamics occurring within the gut, through the life and death of microbes within.

It is likely that many of the positive impacts of microbes upon host biology are correlated consequences of microbial evolution selected for other reasons [110]. Holobiont properties may relate to adaptation of hosts to microbe presence, including coadaptation where microbes are required as signals during development. These types of evolutionary processes provide the appearance of the holobiont as a level of selection, but in fact are underlain by different processes, where selection is occurring on the microbe without reference to host fitness or the host without reference to the microbe fitness.

Finally, while there is much debate in the field associated with terms such as holobiont and hologenome, and whether holobionts are usefully considered a level of selection, this discord in some senses contrasts with a widespread acceptance of the need to understand the complex interplays outlined above in order to establish the evolutionary ecology and dynamics of host–microbe interactions. Many of the features described above are echoed in treatments of hologenomes as they are here (e.g. [19]). It is upon these processes that we must focus our research effort.

## Data accessibility

This article has no additional data.
